# Timing, fractionation, and dose of thoracic radiotherapy in patients with extensive-stage small cell lung cancer undergoing first-line Chemo-Immunotherapy

**DOI:** 10.1016/j.ctro.2026.101114

**Published:** 2026-02-05

**Authors:** Hao Zhou, Huan Zhao, Shuming Shi, Tao Hu, Li Li, Shuai Wang, Zhe Zhang, Shuanghu Yuan

**Affiliations:** a Shandong Cancer Hospital and Institute Shandong First Medical University and Shandong Academy of Medical Sciences, China; b Shandong Provincial Public Health Clinical Center, China; c The First Affiliated Hospital of USTC, Division of Life Sciences and Medicine, University of Science and Technology of China, China; d Peking University Shenzhen Hospital, China

**Keywords:** Small Cell Lung Cancer, Radiation, Immunotherapy

## Abstract

**Objective:**

This study investigates the optimal timing, fractionation, and dose of thoracic radiotherapy (RT) in extensive-stage small cell lung cancer (ES-SCLC) patients responsive to first-line Chemo-Immunotherapy (CIT), and evaluates efficacy and safety.

**Materials and methods:**

In this multicenter retrospective analysis, 280 ES-SCLC patients receiving both CIT and TRT between January 2020 and July 2024 were included. Patients were stratified by TRT timing into Concurrent Chemo-Immuno-Radiotherapy (CCIRT, during CIT) or Sequential CIRT (SCIRT, after CIT) groups. We also evaluated the differences among various prescribed radiation doses and fractionation regimens. To avoid selection bias, we conducted Propensity Score Matching (PSM) for patients. Overall survival (OS), progression-free survival (PFS), and treatment-related adverse events (TRAEs) were assessed and compared.

**Results:**

The median PFS and OS in the study were 8.7 months and 20.6 months, respectively. The SCIRT group demonstrated superior PFS (p = 0.008) and OS (p = 0.048) compared with the CCIRT group, and additionally with a lower incidence of TRAEs. In the CCIRT group, a low time-corrected biological effective dose (tBED) (≤50 Gy) was associated with better OS (p = 0.028) and PFS (p = 0.014). Sex, KPS, tBED, brain metastasis status, and other indicators were independent influencing factors for TRAEs. Age, KPS, tBED, and distant metastasis status were independent prognostic factors.

**Conclusion:**

Based on the efficacy and safety profile, sequential TRT after first-line chemoimmunotherapy is superior to concurrent administration. The exploratory conclusions regarding the optimal dose and fractionation regimen require confirmation through further research.

## Highlights

• The optimal thoracic RT timing for ES-SCLC is controversial. We evaluated early RT, which failed to improve survival but increased toxicity.
• RT dose and fractionation impact ES-SCLC outcomes, affecting tumor control and quality of life. We performed an exploratory analysis.
• Safety is critical in ES-SCLC. We explored toxicity-related factors during TRT to inform individualized clinical management.
• This study identified independent prognostic factors for ES-SCLC patients receiving TRT, enabling early and precise outcome prediction.

## Introduction

SCLC accounts for 10–15% of all lung cancers, with approximately 70% of patients diagnosed at the extensive stage [1]. ES-SCLC is highly aggressive and carries a very poor prognosis. Although ES-SCLC patients typically show high objective response rates to initial systemic therapy, most experience rapid progression or death due to recurrence and metastasis, often with poor control of intrathoracic lesions [2], [3]. Prospective clinical trials such as CASPIAN and IMpower133 have demonstrated that adding immunotherapy significantly improves prognosis in ES-SCLC, establishing it as a first-line treatment [4], [5]. A series of retrospective studies also suggest that administering thoracic radiotherapy to ES-SCLC patients who achieve remission after systemic therapy can improve survival by better controlling intrathoracic lesions [6], [7], [8].

Nevertheless, within the context of immunotherapy, the overall risk–benefit assessment of thoracic radiotherapy for ES-SCLC still requires elucidation through high-quality evidence [9]. It is noteworthy that the baseline condition of ES-SCLC patients is commonly suboptimal, making them more prone to treatment toxicities under intensified or combined modality regimens [10], [11]. A retrospective study has demonstrated that, compared to CIT treatment alone, combined thoracic radiotherapy treatment significantly improves prognosis but also causes more marked TRAEs [12]. Consequently, optimizing treatment strategies necessitates careful weighing of efficacy against toxicity risks. However, studies such as CASPIAN did not include Concurrent Chemoradiotherapy (CCRT); its role in first-line ES-SCLC treatment remains under investigation. A prospective study of 56 patients demonstrated that administering concurrent low-dose RT (LDRT, 15 Gy) during the CIT achieved a mPFS of 6.9 months, and a 12-month OS rate of 71.9% [13]. This study provides preliminary support for the application of CCRT in ES-SCLC, suggesting its potential clinical benefit.

There is currently no definitive consensus regarding the optimal application of thoracic radiotherapy. Therefore, this study aims to investigate the optimal timing of thoracic radiotherapy in patients with ES-SCLC, systematically evaluate the appropriate radiotherapy regimen and doses, identify high-risk groups for adverse reactions, and analyze independent prognostic factors. The results will provide evidence for individualized RT decision-making and help predict treatment efficacy at the initial treatment stage.

## Materials and methods

### Cohort

**Fig. 1:**
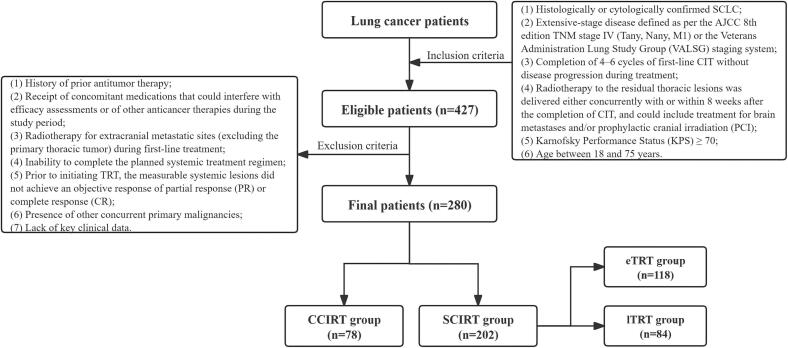
Inclusion and exclusion flowchart.

This multicenter, retrospective cohort study comprised 280 patients with histologically confirmed ES-SCLC who received treatment at Shandong Cancer Hospital, Anhui Provincial Hospital, and Anhui Cancer Hospital between January 2020 and July 2024. Extensive-stage disease was defined as stage IV disease (Tany, Nany, M1) according to the AJCC 8th edition TNM staging system or as per the Veterans Administration Lung Study Group (VALSG) criteria. The inclusion and exclusion criteria are presented in Fig. 1.

All patients received 4–6 cycles of platinum-etoposide chemotherapy combined with immune checkpoint inhibitors (ICIs), and underwent thoracic radiotherapy either concurrently with this first-line treatment or within approximately 8 weeks after completion of systemic therapy. Thoracic radiotherapy was delivered using either 3D conformal radiotherapy (3D-CRT) or intensity-modulated radiation therapy (IMRT). The gross tumor volume (GTV) included the primary tumor and any positive lymph nodes. The clinical target volume (CTV) was defined as the GTV plus a 5-mm margin. An additional margin of 5–8 mm was added to the CTV to generate the planning target volume (PTV). For cases where the tumor volume was too large to create a tolerable plan, a planning gross tumor volume (PGTV) was created by directly expanding the GTV by 5–10 mm. Dose calculation verification was performed for all plans as part of the quality assurance process. Vn represents the percentage of total lung volume which was irradiated to a dose of at least nGy.

We typically use the BED to convert between radiotherapy protocols for estimating biological effects on tissues [14]. Given variations in fractional doses and treatment times across clinical protocols, we applied the tBED equation [15]: tBED = (nd)(1 + d/(α/β)) − (0.693 t/(αTpot)), where n is the number of fractions, d represents the dose per fraction (Gy), α/β = 10 Gy, α = 0.3 Gy, t is the number of radiotherapy days (days), and Tpot is the potential doubling time(5.6 days)[16].

### Efficacy and safety assessment criteria

PFS was defined as the time from the initiation of systemic treatment until the first occurrence of either disease progression or death from any cause. OS was defined as the time from the initiation of systemic treatment to death from any cause. Tumor response was evaluated per Response Evaluation Criteria in Solid Tumors version 1.1 (RECIST v1.1) [17]. Efficacy assessments were performed every 6–8 weeks until disease progression or death. All TRAEs were graded according to the Common Terminology Criteria for Adverse Events (CTCAE) version 5.0 [18].

### Statistical analysis

PSM and inverse probability weighting (IPW) were used to balance baseline covariates. PSM was performed using the nearest-neighbor method with a caliper width of 0.2. The matching ratio (1:1 or 1:N) was determined by the relative size of the patient cohorts. Extreme weights in IPW (below the 1st percentile or above the 99th, or absolute values < 0.1 or > 10) were Winsorized to minimize bias [19]. After matching, the balance between groups was assessed by calculating the standardized mean difference (SMD). An absolute SMD value < 0.1 indicates excellent covariate balance, a value between 0.1 and 0.2 suggests acceptable balance, and a value > 0.2 signals potentially meaningful residual imbalance. Continuous variables were compared using Student’s *t*-test or the Mann–Whitney *U* test, and categorical variables were compared using the Chi-square test or Fisher’s exact test. Independent risk factors for TRAEs were identified via logistic regression. Hazard ratio (HR) for PFS and OS were estimated using Cox models, from which independent prognostic predictors were derived. Survival curves were generated with the Kaplan–Meier method, and compared using log-rank tests and weighted Cox models. The proportional hazards assumption was assessed with Schoenfeld residuals; If violated, time-dependent Cox regression and piecewise Cox regression were employed. For subgroups with fewer than 20 cases, results are presented descriptively only, without formal inferential hypothesis testing. All analyses were conducted using SPSS 27.0 and R 4.5.0.

## Results

### Patient characteristics

This study included a total of 280 patients: 78 in the CCIRT group and 202 in the SCIRT group. In the SCIRT group, 118 patients underwent early TRT (eTRT, within 4 weeks of completing CIT), compared with 84 who received late TRT (lTRT, 4–8 weeks after CIT completion). The majority of patients were male (232/280, 82.9%), and 154 (55.0%) were aged 60 years or older. Distant metastases involving two or more organs were observed in 121 patients (43.2%). 234 patients (83.6%) received > 4 cycles of CIT. Among the 64 patients (22.9%) who developed brain metastases, 52 (81.3%) received radiotherapy for brain metastases during first-line treatment. Reasons for delaying or omitting first-line brain radiotherapy include disease progression, deterioration in KPS, and patient refusal. Prophylactic cranial irradiation (PCI) was administered to 22 patients (7.9%). Maintenance therapy with ICIs following CIT was received by 226 patients (80.7%). Of the 54 patients who did not receive maintenance immunotherapy, reasons included: severe adverse events (SAEs) (23/54, 42.6%), voluntary patient withdrawal (12/54, 22.2%), disease progression (11/54, 20.4%), decline in KPS score (7/54, 13.0%), and death (1/54, 1.9%). Before receiving TRT, all patients had achieved a partial (PR) or complete response (CR) in their systemic lesions. In the SCIRT group, 150 patients (74.3%) achieved PR/CR during first-line systemic therapy, and the remaining 52 (25.8%) did so at the post-therapy assessment. The TRT fractionation regimens consisted of: conventional fractionation radiotherapy (cRT; 46–66 Gy/23–33F/1.8–2.0 Gy; n = 170), hyperfractionated radiotherapy (hyperRT; 45–60 Gy/30–40F/1.5 Gy; n = 51), and hypofractionated radiotherapy (hypoRT; 30–60 Gy/10–24F/2.2–3.0 Gy; n = 59). The median tBED was 47.4 Gy (IQR, 44.3–51.5 Gy) (Table 1).

**Table 1:** Baseline Characteristics of the Patients. Sequential radiotherapy: Patients received thoracic radiotherapy within 8 weeks after completing first-line chemoimmunotherapy. Based on the interval to radiotherapy, they were categorized into two groups using 4 weeks as the cutoff: those with an interval of ≤ 4 weeks and those with an interval of > 4 to ≤ 8 weeks. KPS: Karnofsky Performance Status; CIT: Chemo-Immunotherapy; ICIs: immune checkpoint inhibitors; PCI: Prophylactic Cranial Irradiation.

Variable	Total (n = 280, %)
Timing of Radiotherapy	
Concurrent radiotherapy	78 (27.9)
Sequential radiotherapy ≤ 4 weeks 4–8 weeks	202 (72.1) 118 (42.1) 84 (30.0)
≤4 weeks	118 (42.1)
4–8 weeks	84 (30.0)
Radiotherapy Fractionation Scheme	
conventional fractionation radiotherapy	170 (60.7)
hyperfractionated radiotherapy	51 (18.2)
hypofractionated radiotherapy	59 (20.1)
Age (years)	
<60	126 (45.0)
≥60	154 (55.0)
Sex	
female	48 (17.1)
male	232 (82.9)
Smoking	
No	101 (36.1)
Yes	179 (63.9)
Kps	
≤80	128 (45.7)
>80	152 (54.3)
Family history of cancer	
No	231 (82.5)
Yes	49 (17.5)
Weight loss	
No	236 (84.3)
Yes	44 (15.7)
T stage	
≤2	127 (45.4)
>2	153 (54.6)
N stage	
0	3 (1.1)
1	20 (7.1)
2	123 (43.9)
3	134 (47.9)
Brain metastasis	
No	216 (77.1)
Yes	64 (22.9)
Liver metastasis	
No	221 (78.9)
Yes	59 (21.1)
Bone metastasis	
No	216 (77.1)
Yes	64 (22.9)
Number of metastasis organs	
<2	159 (56.8)
≥2	121 (43.2)
CIT cycles	
4	46 (16.4)
5	45 (16.1)
6	189 (67.5)
Types of ICIs	
PD-1	75 (26.8)
PD-L1	205 (73.2)
Radiation dose (Gy)	
≤50	157 (56.1)
>50	123 (43.9)
Radiotherapy for brain metastases	
No	228 (81.4)
Yes	52 (18.6)
PCI	
No	258 (92.1)
Yes	22 (7.9)

### Survival outcomes and adverse events

Of the entire study population, 253 patients (90.4%) experienced disease progression. Among these, 49 (19.4%) had localized progression in the chest, 172 (68.0%) had distant progression, and 32 (12.6%) had both. The mPFS was 8.7 months, with PFS rates of 81.1% at 6 months and 26.1% at 12 months. A total of 185 patients (66.1%) died. The mOS was 20.6 months, and the OS rates were 82.9% at 12 months and 33.2% at 24 months. The median follow-up time was 32.9 months.

Analysis of TRAEs showed that grade ≥ 2 TRAEs occurred in 150 patients (53.6%), including 50 in the CCIRT group and 100 in the SCIRT group. The most common grade ≥ 2 TRAEs were hematologic toxicity, pneumonia, gastrointestinal toxicity, and radiation esophagitis. Grade ≥ 3 TRAEs were observed in 76 patients (27.1%). We defined the following as treatment-modifying adverse events (TMAEs): grade ≥ 2 pneumonia, grade ≥ 3 hematologic toxicity, grade ≥ 3 radiation esophagitis, and grade ≥ 2 hepatitis. These specific events were selected because they often require interruption of antineoplastic therapy, which may impact treatment efficacy (Due to their low severity, which did not affect treatment, or their low frequency, TRAEs such as skin toxicity, fatigue, and gastrointestinal toxicity were omitted from the analysis). TMAEs occurred in 35 patients in the CCIRT group and 58 in the SCIRT group. Treatment interruptions due to TMAEs occurred in 72 patients (77.4%), primarily because of hematologic toxicity or pneumonia (Table 2).

**Table 2:** Summary Table of Treatment-Related Adverse Events. CCIRT: Concurrent Chemo-Immuno-Radiotherapy; SCIRT: Sequential Chemo-Immuno-Radiotherapy; TRAEs: Treatment-related adverse reactions. TMAEs: Treatment-modifying adverse events, which refer to grade ≥ 2 pneumonitis, grade ≥ 3 hematologic toxicity, grade ≥ 3 radiation esophagitis, or grade ≥ 2 hepatitis.

Adverse Events	CCIRT group (n = 78, %)				SCIRT group (n = 202, %)		
	conventional fractionation radiotherapy (n = 39)	hyperfractionated radiotherapy (n = 28)	hypofractionated radiotherapy (n = 11)		conventional fractionation radiotherapy (n = 131)	hyperfractionated radiotherapy (n = 23)	hypofractionated radiotherapy (n = 48)
TRAEs (grade ≥ 2)	24 (61.5%)	18 (64.3%)	8 (72.7%)		64 (48.9%)	10 (43.5%)	26 (54.2%)
Hematologic toxicity	18 (75.0%)	14 (77.8%)	7 (87.5%)		29 (45.3%)	5 (50.0%)	15 (57.7%)
Pneumonia	8 (33.3%)	6 (33.3%)	3 (37.5%)		24 (37.5%)	4 (40.0%)	9 (34.6%)
Radiation esophagitis	5 (20.8%)	2 (11.1%)	1 (12.5%)		9 (14.1%)	3 (30.0%)	6 (23.1%)
Hepatitis	1 (4.2%)	0 (0.0%)	0 (0.0%)		2 (3.1%)	0 (0.0%)	0 (0.0%)
Gastrointestinal toxicity	11 (45.8%)	8 (44.4%)	4 (50.0%)		23 (35.9%)	4 (40.0%)	10 (38.5%)
Fatigue	1 (4.2%)	0 (0.0%)	1 (12.5%)		4 (6.3%)	1 (10.0%)	1 (3.8%)
Radiation Dermatitis	2 (8.3%)	0 (0.0%)	0 (0.0%)		3 (4.7%)	0 (0.0%)	0 (0.0%)

TMAEs	17 (43.6%)	11 (39.3%)	7 (63.6%)		37 (28.2%)	6 (26.1%)	15 (31.3%)
Hematologic toxicity	11 (64.7%)	6 (54.5%)	6 (85.7%)		15 (40.5%)	4 (66.7%)	11 (73.3%)
Pneumonia	8 (47.1%)	6 (54.5%)	3 (42.9%)		24 (64.9%)	5 (83.3%)	9 (60.0%)
Radiation esophagitis	3 (17.6%)	1 (9.1%)	1 (14.3%)		5 (13.5%)	2 (33.3%)	3 (20.0%)
Hepatitis	1 (5.9%)	0 (0.0%)	0 (0.0%)		2 (5.4%)	0 (0.0%)	0 (0.0%)

### Comparison of the efficacy and safety of different radiotherapy timings

**Fig. 2:**
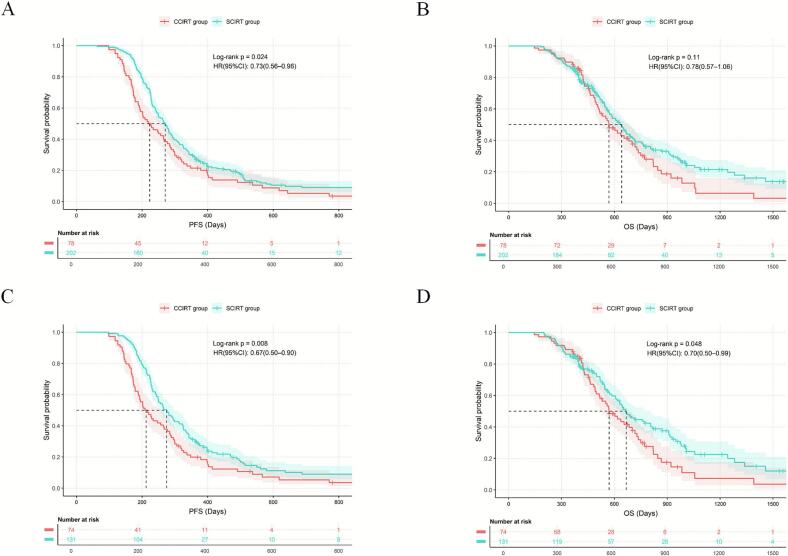
Kaplan–Meier curves for PFS and OS. **(A)** PFS in the CCIRT and SCIRT groups before PSM. **(B)** OS in the CCIRT and SCIRT groups before PSM. **(C)** PFS in the CCIRT and SCIRT groups after PSM. **(D)** OS in the CCIRT and SCIRT groups after PSM.

Following 1:2 PSM, 74 patients in the CCIRT group and 131 in the SCIRT group were included (Table 3). After matching, mPFS was 7.0 months versus 9.2 months, and mOS was 19.0 months versus 22.2 months, respectively. Compared with CCIRT group, SCIRT group showed significantly improved PFS (HR = 0.67, 95%CI: 0.50–0.90, p = 0.008) and OS (HR = 0.70, 95%CI: 0.50–0.99, p = 0.048) (Fig. 2). The probability of locoregional progression at the time of disease progression was 26.1% (18/69) and 33.3% (39/117) in the two groups, respectively (p = 0.30). Grade ≥ 2 TRAEs occurred in 47 (63.5%) CCIRT patients compared with 66 (50.4%) SCIRT patients (p = 0.069), while TMAEs occurred in 33 (44.6%) versus 40 (30.5%) patients (p = 0.043).

**Table 3:** Baseline characteristics of the CCIRT and SCIRT groups before and after PSM (PSM was performed in a 1:2 ratio with a caliper width of 0.2). PSM, propensity score matching; CCIRT: Concurrent Chemo-Immuno-Radiotherapy; SCIRT: Sequential Chemo-Immuno-Radiotherapy; SMD: Standardized Mean Difference; KPS: Karnofsky Performance Status; CIT: Chemo-Immunotherapy; ICIs: immune checkpoint inhibitors; PCI: Prophylactic Cranial Irradiation.

Variable		Before PSM					After PSM			
		SCIRT (n = 202)	CCIRT (n = 78)	P	SMD		SCIRT (n = 131)	CCIRT (n = 74)	P	SMD
Age (years)	<60	89(44.06)	37(47.44)	0.611	0.068		60(45.80)	35(47.30)	0.837	0.030
	≥60	113(55.94)	41(52.56)		−0.068		71(54.20)	39(52.70)		−0.030

Sex	female	32(15.84)	16(20.51)	0.352	0.116		22(16.79)	15(20.27)	0.534	0.086
	male	170(84.16)	62(79.49)		−0.116		109(83.21)	59(79.73)		−0.086

Smoking	No	71(35.15)	30(38.46)	0.605	0.068		47(35.88)	27(36.49)	0.931	0.013
	Yes	131(64.85)	48(61.54)		−0.068		84(64.12)	47(63.51)		−0.013

Kps	≤80	90(44.55)	38(48.72)	0.531	0.083		60(45.80)	35(47.30)	0.837	0.030
	>80	112(55.45)	40(51.28)		−0.083		71(54.20)	39(52.70)		−0.030

Family history of cancer	No	165(81.68)	66(84.62)	0.563	0.081		108(82.44)	62(83.78)	0.806	0.036
	Yes	37(18.32)	12(15.38)		−0.081		23(17.56)	12(16.22)		−0.036

Weight loss	No	168(83.17)	68(87.18)	0.408	0.120		112(85.50)	64(86.49)	0.845	0.029
	Yes	34(16.83)	10(12.82)		−0.120		19(14.50)	10(13.51)		−0.029

T stage	≤2	91(45.05)	36(46.15)	0.868	0.022		64(48.85)	35(47.30)	0.830	−0.031
	>2	111(54.95)	42(53.85)		−0.022		67(51.15)	39(52.70)		0.031

N stage	0	2(0.99)	1(1.28)	0.196	0.026		1(0.76)	1(1.35)	0.676	0.051
	1	17(8.42)	3(3.85)		−0.238		4(3.05)	3(4.05)		0.051
	2	82(40.59)	41(52.56)		0.240		59(45.04)	38(51.35)		0.126
	3	101(50.00)	33(42.31)		−0.156		67(51.15)	32(43.24)		−0.159

Brain metastasis	No	155(76.73)	61(78.21)	0.793	0.036		101(77.10)	57(77.03)	0.991	−0.002
	Yes	47(23.27)	17(21.79)		−0.036		30(22.90)	17(22.97)		0.002

Liver metastasis	No	157(77.72)	64(82.05)	0.426	0.113		106(80.92)	60(81.08)	0.977	0.004
	Yes	45(22.28)	14(17.95)		−0.113		25(19.08)	14(18.92)		−0.004

Bone metastasis	No	157(77.72)	59(75.64)	0.710	−0.048		102(77.86)	55(74.32)	0.566	−0.081
	Yes	45(22.28)	19(24.36)		0.048		29(22.14)	19(25.68)		0.081

Number of metastasis organs	<2	110(54.46)	49(62.82)	0.205	0.173		75(57.25)	45(60.81)	0.619	0.073
	≥2	92(45.54)	29(37.18)		−0.173		56(42.75)	29(39.19)		−0.073

CIT cycles	4	33(16.34)	13(16.67)	0.249	0.009		23(17.56)	13(17.57)	0.990	0.000
	5	28(13.86)	17(21.79)		0.192		22(16.79)	13(17.57)		0.020
	6	141(69.80)	48(61.54)		−0.170		86(65.65)	48(64.86)		−0.016

Types of ICIs	PD-1	53(26.24)	22(28.21)	0.739	0.044		37(28.24)	20(27.03)	0.852	−0.027
	PD-L1	149(73.76)	56(71.79)		−0.044		94(71.76)	54(72.97)		0.027

Radiation dose (Gy)	≤50	115(56.93)	42(53.85)	0.641	−0.062		71(54.20)	39(52.70)	0.837	−0.030
	>50	87(43.07)	36(46.15)		0.062		60(45.80)	35(47.30)		0.030

Radiotherapy for brain metastases	No	163(80.69)	65(83.33)	0.611	0.071		108(82.44)	61(82.43)	0.999	−0.000
	Yes	39(19.31)	13(16.67)		−0.071		23(17.56)	13(17.57)		0.000

PCI	No	189(93.56)	69(88.46)	0.155	−0.160		121(92.37)	66(89.19)	0.440	−0.102
	Yes	13(6.44)	9(11.54)		0.160		10(7.63)	8(10.81)		0.102

**Fig. 3:**
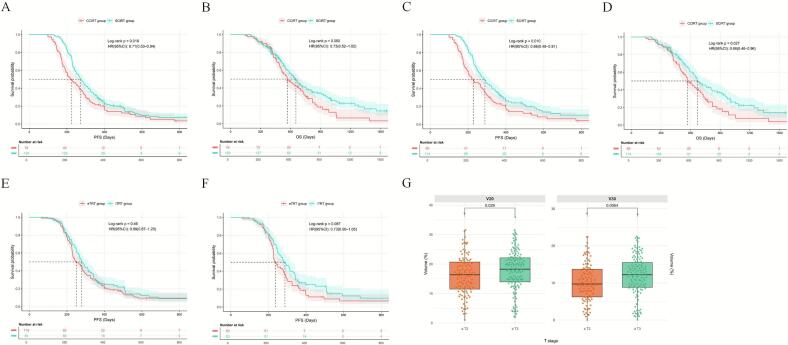
Analysis of Patients Achieving PR/CR During CIT and of Early vs. Late Sequential Radiotherapy. **(A)** PFS in the CCIRT and SCIRT groups before PSM. **(B)** OS in the CCIRT and SCIRT groups before PSM. **(C)** PFS in the CCIRT and SCIRT groups after PSM. **(D)** OS in the CCIRT and SCIRT groups after PSM. **(E)** PFS in the eTRT and lTRT groups before PSM. **(F)** PFS in the eTRT and lTRT groups after PSM. **(G)** Differences in lung V20 and V30 between patient groups by T stage (≤2 versus ≥ 3).

After 1:2 PSM, supplementary analyses showed that even among patients who achieved PR or CR during first-line immunotherapy, the SCIRT group maintained superior PFS (HR = 0.66, 95%CI: 0.48–0.91; p = 0.010) and OS (HR = 0.66, 95%CI: 0.46–0.96; p = 0.027) (Table 4 and Fig. 3). After 1:1 PSM, compared to the eTRT subgroup, the lTRT subgroup showed a trend toward longer PFS (p = 0.087), and no statistically significant differences in TRAEs were observed between the two groups (Fig. 3).

**Table 4:** Baseline characteristics of the CCIRT and SCIRT groups before and after PSM, in the patient cohort achieving PR/CR during first-line therapy (PSM was performed in a 1:2 ratio with a caliper width of 0.2). PSM, Propensity Score Matching; CCIRT: Concurrent Chemo-Immuno-Radiotherapy; SCIRT: Sequential Chemo-Immuno-Radiotherapy; SMD: Standardized Mean Difference; KPS: Karnofsky Performance Status; CIT: Chemo-Immunotherapy; ICIs: immune checkpoint inhibitors; PCI: Prophylactic Cranial Irradiation.

Variable		Before PSM					After PSM			
		SCIRT (n = 150)	CCIRT (n = 78)	P	SMD		SCIRT (n = 114)	CCIRT (n = 69)	P	SMD
Age (years)	<60	70(46.67)	37(47.44)	0.912	0.015		53(46.49)	30(43.48)	0.692	−0.061
	≥60	80(53.33)	41(52.56)		−0.015		61(53.51)	39(56.52)		0.061

Sex	female	24(16.00)	16(20.51)	0.395	0.112		17(14.91)	11(15.94)	0.851	0.028
	male	126(84.00)	62(79.49)		−0.112		97(85.09)	58(84.06)		−0.028

Smoking	No	44(29.33)	30(38.46)	0.163	0.188		36(31.58)	23(33.33)	0.806	0.037
	Yes	106(70.67)	48(61.54)		−0.188		78(68.42)	46(66.67)		−0.037

Kps	≤80	59(39.33)	38(48.72)	0.174	0.188		49(42.98)	31(44.93)	0.797	0.039
	>80	91(60.67)	40(51.28)		−0.188		65(57.02)	38(55.07)		−0.039

Family history of cancer	No	122(81.33)	66(84.62)	0.536	0.091		96(84.21)	59(85.51)	0.813	0.037
	Yes	28(18.67)	12(15.38)		−0.091		18(15.79)	10(14.49)		−0.037

Weight loss	No	120(80.00)	68(87.18)	0.176	0.215		99(86.84)	60(86.96)	0.982	0.003
	Yes	30(20.00)	10(12.82)		−0.215		15(13.16)	9(13.04)		−0.003

T stage	≤2	59(39.33)	36(46.15)	0.322	0.137		50(43.86)	35(50.72)	0.367	0.137
	>2	91(60.67)	42(53.85)		−0.137		64(56.14)	34(49.28)		−0.137

N stage	0	2(1.33)	1(1.28)	0.142	−0.005		1(0.88)	1(1.45)	0.749	0.048
	1	13(8.67)	3(3.85)		−0.251		5(4.39)	3(4.35)		−0.002
	2	57(38.00)	41(52.56)		0.292		50(43.86)	35(50.72)		0.137
	3	78(52.00)	33(42.31)		−0.196		58(50.88)	30(43.48)		−0.149

Brain metastasis	No	109(72.67)	61(78.21)	0.362	0.134		87(76.32)	52(75.36)	0.884	−0.022
	Yes	41(27.33)	17(21.79)		−0.134		27(23.68)	17(24.64)		0.022

Liver metastasis	No	122(81.33)	59(75.64)	0.313	−0.133		90(78.95)	51(73.91)	0.433	−0.115
	Yes	28(18.67)	19(24.36)		0.133		24(21.05)	18(26.09)		0.115

Bone metastasis	No	114(76.00)	64(82.05)	0.295	0.158		93(81.58)	56(81.16)	0.944	−0.011
	Yes	36(24.00)	14(17.95)		−0.158		21(18.42)	13(18.84)		0.011

Number of metastasis organs	<2	83(55.33)	49(62.82)	0.277	0.155		65(57.02)	41(59.42)	0.750	0.049
	≥2	67(44.67)	29(37.18)		−0.155		49(42.98)	28(40.58)		−0.049

CIT cycles	4	17(11.33)	13(16.67)	0.036	0.143		14(12.28)	13(18.84)	0.236	0.168
	5	17(11.33)	17(21.79)		0.253		17(14.91)	14(20.29)		0.134
	6	116(77.33)	48(61.54)		−0.325		83(72.81)	42(60.87)		−0.245

Types of ICIs	PD-1	40(26.67)	22(28.21)	0.804	0.034		32(28.07)	18(26.09)	0.770	−0.045
	PD-L1	110(73.33)	56(71.79)		−0.034		82(71.93)	51(73.91)		0.045

Radiation dose (Gy)	≤50	84(56.00)	42(53.85)	0.756	−0.043		66(57.89)	39(56.52)	0.856	−0.028
	>50	66(44.00)	36(46.15)		0.043		48(42.11)	30(43.48)		0.028

Radiotherapy for brain metastases	No	123(82.00)	65(83.33)	0.802	0.036		92(80.70)	56(81.16)	0.939	0.012
	Yes	27(18.00)	13(16.67)		−0.036		22(19.30)	13(18.84)		−0.012

PCI	No	143(95.33)	69(88.46)	0.054	−0.215		107(93.86)	63(91.30)	0.722	−0.091
	Yes	7(4.67)	9(11.54)		0.215		7(6.14)	6(8.70)		0.091

In the CCIRT group, PD-L1 Inhibitor (OR = 0.14, 95%CI: 0.03–0.59, p = 0.007) and T stage (≥T3; OR = 5.28, 95%CI: 1.74–16.01, p = 0.003) were independent influencing factors for grade ≥ 2 TRAEs, while KPS (>80; OR = 0.16, 95%CI: 0.05–0.51, p = 0.002), tBED (>50 Gy; OR = 4.84, 95%CI: 1.48–15.81, p = 0.009), and baseline brain metastasis (OR = 4.43, 95%CI: 1.18–16.61, p = 0.027) were independent influencing factors for TMAEs. In the SCIRT group, sex (male; OR = 0.33, 95%CI: 0.12–0.92, p = 0.035) and T stage (≥T3; OR = 2.40, 95%CI: 1.17–4.92, p = 0.016) were independent influencing predictors for grade ≥ 2 TRAEs, while smoking history (OR = 2.59, 95%CI: 1.08–6.21, p = 0.032) and weight loss (OR = 3.23, 95%CI: 1.16–8.98, p = 0.025) were independent influencing predictors for TMAEs. Patients with T stage ≥ 3 had significantly higher lung V20 (18.26% versus 16.36%; p = 0.029) and V30 (12.21% versus 9.72%; p = 0.006) than those with T stage ≤ 2 (Fig. 3).

### Comparative efficacy and safety of different fractionation schedules and doses

**Fig. 4:**
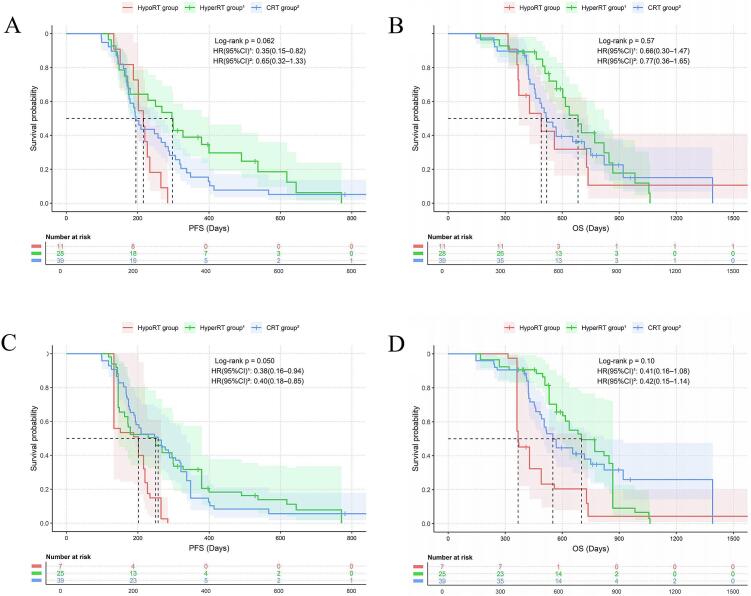
PFS and OS by the fractionation regimen in the CCIRT group. **(A)** PFS before IPW. **(B)** OS before IPW. **(C)** PFS after IPW. **(D)** OS after IPW.

**Fig. 5:**
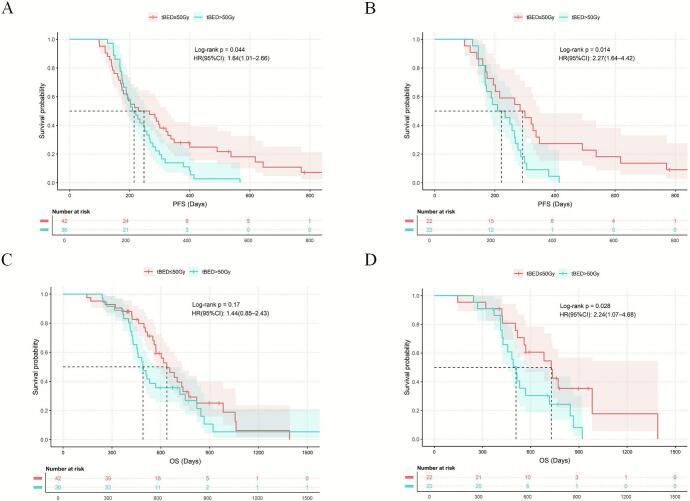
PFS and OS by radiotherapy dose group in the CCIRT group. **(A)** PFS before PSM. **(B)** PFS after PSM. **(C)** OS before PSM. **(D)** OS after PSM.

In the CCIRT group, inverse probability weighting analysis revealed that non-hypoRT (including cRT and hyperRT) was associated with numerically longer PFS compared to hypoRT (p = 0.050; Table 5 and Fig. 4), along with a trend towards improved OS (p = 0.10). No statistically significant difference in the incidence of adverse events was observed among the three patient groups. However, the statistical power of this comparison was limited by the small sample size. In the SCIRT group, no significant differences in prognosis or adverse events were observed among the three radiotherapy modalities. Regarding radiation dose, following 1:1 propensity score matching, a high tBED (>50 Gy) within the CCIRT group was associated with significantly worse OS (HR = 2.24, 95% CI: 1.07–4.68, p = 0.028) and PFS (HR = 2.27, 95% CI: 1.64–4.42, p = 0.014), compared with a low tBED (≤50 Gy) (Fig. 5). The incidence of TMAEs differed significantly between the two groups (63.6% versus 31.8%, p = 0.035). In the SCIRT group after 1:1 PSM, the incidence of TMAEs was also higher in the high-tBED group than in the low-tBED group (36.7% vs. 20.3%, p = 0.022) (Table 6).

**Table 5:** Baseline characteristics of the hypoRT, hyperRT, and cRT groups, before and after IPW. Variable selection was based on a priori clinical knowledge and univariable screening. Given the sample size limitations, LASSO regression was not feasible. IPW: inverse probability weighting; HypoRT: hypofractionated radiotherapy; HyperRT: hyperfractionated radiotherapy; CRT: conventional radiotherapy; SMD: Standardized Mean Difference; KPS: Karnofsky Performance Status. CIT: Chemo-Immunotherapy; ICIs: immune checkpoint inhibitors; PCI: Prophylactic Cranial Irradiation.

Variable		Before IPW				After IPW			
		HypoRT group (n = 11)	HyperRT group (n = 28)	CRT group (n = 39)	|SMD|	HypoRT group (n = 7.3)	HyperRT group (n = 24.9)	CRT group (n = 38.8)	|SMD|
Age (years)	<60	6(54.55)	12(42.86)	19(48.72)	0.157	2.2(30.60)	10.5(38.80)	18.2(47.00)	0.083
	≥60	5(45.45)	16(57.14)	20(51.28)		5.1(69.40)	14.4(61.20)	20.6(53.00)	

Sex	female	0(0.00)	6(21.43)	10(25.64)	0.556	0.0(0.00)	8.7(35.10)	7.5(19.40)	0.157
	male	11(100.0)	22(78.57)	29(74.36)		8.0(100.00)	16.2(64.90)	31.3(80.60)	

Smoking	No	3(27.27)	12(42.86)	15(38.46)	0.220	1.0(13.20)	11.6(46.60)	12.3(31.80)	0.148
	Yes	8(72.73)	16(57.14)	24(61.54)		6.3(86.80)	13.3(53.40)	26.5(68.20)	

Kps	≤80	6(54.55)	12(42.86)	20(51.28)	0.157	3.0(40.60)	8.3(33.30)	17.1(44.10)	0.108
	>80	5(45.45)	16(57.14)	19(48.72)		4.3(59.40)	16.6(66.70)	21.7(55.90)	

Weight loss	No	9(81.82)	25(89.29)	34(87.18)	0.143	5.2(70.70)	15.6(62.50)	25.1(64.70)	0.038
	Yes	2(18.18)	3(10.71)	5(12.82)		2.1(29.30)	9.3(37.50)	13.7(35.30)	

T stage	≤2	6(54.55)	11(39.29)	19(48.72)	0.206	2.3(31.50)	12.4(49.9)	19.1(49.20)	0.007
	>2	5(45.45)	17(60.71)	20(51.28)		5.0(68.50)	12.5(50.10)	19.7(50.80)	

Brain metastasis	No	8(72.73)	24(85.71)	29(74.36)	0.216	5.3(73.10)	21.2(85.00)	29.5(76.10)	0.089
	Yes	3(27.27)	4(14.29)	10(25.64)		2.0(26.90)	3.7(15.0)	9.3(23.90)	

Liver metastasis	No	5(45.45)	27(96.43)	32(82.05)	0.886	4.5(61.20)	23.6(94.68)	31.9(82.20)	0.125
	Yes	6(54.55)	1(3.57)	7(17.95)		2.8(38.80)	1.3(5.32)	6.9(17.80)	

Bone metastasis	No	5(45.45)	25(89.29)	29(74.36)	0.690	5.7(78.00)	18.2(73.10)	30.1(77.60)	0.045
	Yes	6(54.55)	3(10.71)	10(25.64)		1.6(22.00)	6.7(26.90)	8.7(22.40)	

Number of metastasis organs	<2	4(36.36)	22(78.57)	23(58.97)	0.614	5.2(70.70)	15.6(62.50)	25.1(64.70)	0.023
	≥2	7(63.64)	6(21.43)	16(41.03)		2.1(29.30)	9.3(37.50)	13.7(35.30)	

CIT cycles	4	1(9.09)	4(14.29)	8(20.51)	0.218	3.2(44.10)	6.4(25.90)	6.2(16.00)	0.099
	≥4	10(90.91)	24(85.71)	31(79.49)		4.1(55.90)	18.5(74.10)	32.6(84.00)	

Types of ICIs	PD-1	2(18.18)	9(32.14)	11(28.21)	0.217	1.1(14.60)	5.9(23.70)	11.8(30.40)	0.067
	PD-L1	9(81.82)	19(67.86)	28(71.79)		6.2(85.40)	19.0(76.30)	27.0(69.60)	

Radiation dose (Gy)	≤50	4(36.36)	22(78.57)	16(41.03)	0.623	4.5(61.60)	13.3(53.60)	21.4(55.20)	0.017
	>50	7(63.64)	6(21.43)	23(58.97)		2.8(38.40)	11.6(46.40)	17.4(44.80)

**Table 6:** Baseline characteristics of the low-tBED and high-tBED groups before and after PSM in the CCIRT group (PSM was performed in a 1:1 ratio with a caliper width of 0.2). PSM, Propensity Score Matching; tBED: time-corrected Biological Effective Dose; Low-tBED: ≤50 Gy; High-tBED: >50 Gy; SMD: Standardized Mean Difference; CRT: conventional radiotherapy; HyperRT: hyperfractionated radiotherapy; HypoRT: hypofractionated radiotherapy; KPS: Karnofsky Performance Status; CIT: Chemo-Immunotherapy; ICIs: immune checkpoint inhibitors; PCI: Prophylactic Cranial Irradiation.

Variable		Before PSM				After PSM			
		Low-tBED (n = 42)	High-tBED (n = 36)	P	SMD	Low-tBED (n = 22)	High-tBED (n = 22)	P	SMD
Radiation Fractionation	CRT	16(38.10)	23(63.89)	0.004	0.537	13(59.09)	13(59.09)	1.000	0.000
	HyperRT	22(52.38)	6(16.67)		−0.958	6(27.27)	6(27.27)		0.000
	Hypo RT	4(9.52)	7(19.44)		0.251	3(13.64)	3(13.64)		0.000

Age (years)	<60	20(47.62)	17(47.22)	0.972	−0.008	10(45.45)	11(50.00)	0.763	0.091
	≥60	22(52.38)	19(52.78)		0.008	12(54.55)	11(50.00)		−0.091

Sex	female	7(16.67)	9(25.00)	0.364	0.192	5(22.73)	4(18.18)	1.000	−0.118
	male	35(83.33)	27(75.00)		−0.192	17(77.27)	18(81.82)		0.118

Smoking	No	15(35.71)	15(41.67)	0.590	0.121	7(31.82)	7(31.82)	1.000	0.000
	Yes	27(64.29)	21(58.33)		−0.121	15(68.18)	15(68.18)		0.000

Kps	≤80	23(54.76)	15(41.67)	0.249	−0.266	12(54.55)	10(45.45)	0.546	−0.183
	>80	19(45.24)	21(58.33)		0.266	10(45.45)	12(54.55)		0.183

Family history of cancer	No	34(80.95)	32(88.89)	0.333	0.253	19(86.36)	18(81.82)	1.000	−0.118
	Yes	8(19.05)	4(11.11)		−0.253	3(13.64)	4(18.18)		0.118

Weight loss	No	38(90.48)	30(83.33)	0.548	−0.192	19(86.36)	19(86.36)	1.000	0.000
	Yes	4(9.52)	6(16.67)		0.192	3(13.64)	3(13.64)		0.000

T stage	≤2	16(38.10)	20(55.56)	0.123	0.351	9(40.91)	9(40.91)	1.000	0.000
	>2	26(61.90)	16(44.44)		−0.351	13(59.09)	13(59.09)		0.000

N stage	<2	2(4.76)	2(5.56)	1.000	0.035	1(4.55)	0(0.00)	1.000	−0.309
	≥2	40(95.24)	34(94.44)		−0.035	21(95.45)	22(100.00)		0.309

Brain metastasis	No	33(78.57)	28(77.78)	0.933	−0.019	18(81.82)	18(81.82)	1.000	0.000
	Yes	9(21.43)	8(22.22)		0.019	4(18.18)	4(18.18)		0.000

Liver metastasis	No	35(83.33)	29(80.56)	0.750	−0.070	16(72.73)	17(77.27)	0.728	0.108
	Yes	7(16.67)	7(19.44)		0.070	6(27.27)	5(22.73)		−0.108

Bone metastasis	No	31(73.81)	28(77.78)	0.684	0.095	15(68.18)	16(72.73)	0.741	0.102
	Yes	11(26.19)	8(22.22)		−0.095	7(31.82)	6(27.27)		−0.102

Number of metastasis organs	<2	30(71.43)	19(52.78)	0.089	−0.374	13(59.09)	12(54.55)	0.761	−0.091
	≥2	12(28.57)	17(47.22)		0.374	9(40.91)	10(45.45)		0.091

CIT cycles	4	8(19.05)	5(13.89)	0.542	−0.149	3(13.64)	4(18.18)	1.000	0.118
	≥4	34(80.95)	31(86.11)		0.149	19(86.36)	18(81.82)		−0.118

Types of ICIs	PD-1	13(30.95)	9(25.00)	0.560	−0.137	4(18.18)	6(27.27)	0.472	0.204
	PD-L1	29(69.05)	27(75.00)		0.137	18(81.82)	16(72.73)		−0.204

Radiotherapy for brain metastases	No	36(85.71)	29(80.56)	0.542	−0.130	19(86.36)	19(86.36)	1.000	0.000
	Yes	6(14.29)	7(19.44)		0.130	3(13.64)	3(13.64)		0.000

PCI	No	36(85.71)	33(91.67)	0.642	0.215	19(86.36)	19(86.36)	1.000	0.000
	Yes	6(14.29)	3(8.33)		−0.215	3(13.64)	3(13.64)		0.000

### Prognostic factors for survival in ES-SCLC

**Fig. 6:**
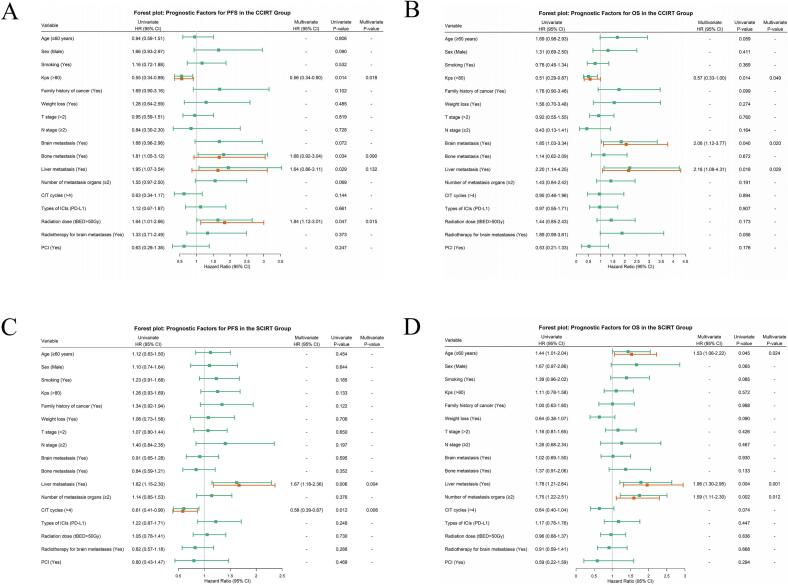
Independent predictors of PFS and OS in the CCIRT and SCIRT groups. **(A)** PFS, CCIRT group. **(B)** OS, CCIRT group. **(C)** PFS, SCIRT group. **(D)** OS, SCIRT group.

In the CCIRT group, KPS (>80; HR = 0.56, 95%CI: 0.34–0.90, p = 0.018) and tBED (>50 Gy; HR = 1.84, 95%CI: 1.12–3.01, p = 0.015) were identified as independent prognostic factors for PFS, while KPS (>80; HR = 0.57, 95%CI: 0.33–1.00, p = 0.049), baseline brain metastasis (HR = 2.06, 95%CI: 1.12–3.77, p = 0.020), and liver metastasis (HR = 2.16, 95%CI: 1.08–4.31, p = 0.029) emerged as independent predictors for OS (Fig. 6).

In the SCIRT group, CIT cycles (>4 cycles; HR = 0.59, 95%CI: 0.39–0.87, p = 0.008) and baseline liver metastasis (HR = 1.67, 95%CI: 1.18–2.36, p = 0.004) were independent prognostic factors for PFS, while age (≥60 years; HR = 1.53, 95%CI: 1.06–2.22, p = 0.024), baseline liver metastasis (HR = 1.96, 95%CI: 1.30–2.95, p = 0.001), and multi-organ metastases (≥2 sites; HR = 1.59, 95%CI: 1.11–2.30, p = 0.012) were independent prognostic factors for OS (Fig. 6).

## Discussion

This multicenter cohort study of 280 patients with ES-SCLC receiving Chemo-Immunotherapy (CIT) and thoracic radiotherapy provides the first systematic comparison of the efficacy and safety between concurrent and sequential thoracic radiotherapy. These findings primarily demonstrate the efficacy, safety, and prognostic factors of the two treatment timings. Furthermore, exploratory analyses of different radiation doses and treatment modalities were conducted. Although statistically underpowered due to limited data, these analyses offer preliminary insights that could guide future research in precision radiotherapy.

Currently, evidence supporting the strategy of concurrent radiotherapy during first-line systemic therapy remains limited in patients with ES-SCLC. Our results showed that concurrent thoracic radiotherapy did not provide superior survival benefits, consistent with the findings of Luo et al. [20]. Specifically, TRAEs may undermine survival benefits by disrupting treatment continuity [21], [22], highlighting the need for optimized toxicity management in combination regimens. For patients in generally good condition (and thus with relatively good tolerance), concurrent radiotherapy during systemic therapy may be a viable option; however, its application should be determined by comprehensively considering patient preferences and the overall treatment strategy [23]. The ongoing phase II trial by Yu et al. (NCT06768307) aims to evaluate the efficacy of CIT administered concurrently versus sequentially with thoracic radiotherapy as first-line treatment for ES-SCLC. This study may offer further high-level evidence to guide thoracic radiotherapy integration in ES-SCLC. Delayed sequential radiotherapy showed a trend toward prolonged PFS and was associated with a lower incidence of treatment-related adverse events. We speculate that one potential mechanism for this survival benefit may be that delaying radiotherapy provides a more optimal window for toxicity management. This helps reduce toxicity-driven treatment interruptions, thereby ensuring the complete delivery of the intended treatment protocol [24].

Prior studies confirm low-dose radiotherapy offers definite efficacy with manageable toxicity [13]. This study found that patients receiving higher-dose thoracic radiotherapy (tBED > 50 Gy) had poorer survival, suggesting the risk–benefit profile of high-dose radiotherapy in ES-SCLC needs re-evaluation. First, since the disease is systemic, further local dose escalation may have reached a therapeutic plateau, limiting additional survival benefit [25]. Second, increased toxicity from higher doses can lead to treatment interruptions, offsetting potential advantages in local control and undermining overall treatment balance [26]. Additionally, patient selection bias must be noted: those with larger initial tumors or a poor response to first-line therapy—often indicating more aggressive disease—are also more likely to receive higher radiotherapy doses. Thus, a high dose may itself be a marker of aggressive biology. Therefore, based on this study's data, routine high-dose thoracic radiotherapy is not recommended for ES-SCLC. To better balance local control and systemic therapy tolerance, adopting moderate-dose regimens (e.g., tBED ≤ 50 Gy, as in 56 Gy/28F, 54 Gy/36F, or 45 Gy/15F) is preferable. This conclusion remains exploratory and requires validation in future large-scale studies that better control for confounding factors. Analysis of radiotherapy fractionation patterns indicated that within the Concurrent Chemo-Immuno-Radiotherapy (CCIRT) group, patients who received non-hypofractionated radiotherapy showed a numerically longer PFS. However, this comparison was statistically underpowered due to the limited sample size. Therefore, this finding should be considered a preliminary hypothesis that requires validation in large-scale prospective studies. In contrast, the three fractionation regimens in the Sequential Chemo-Immuno-Radiotherapy (SCIRT) group showed comparable efficacy, consistent with previous findings [27], suggesting that fractionation patterns may not be the primary factor influencing prognosis in this population or treatment setting.

Safety remains critical in combined cancer therapy. This study reported a 27.1% incidence of grade ≥ 3 AEs, higher than the 10.5% (26/247) in the CREST trial with 30 Gy/10 fractions [28]. This difference may stem from ICIs use and dose-dependent toxicity, as many patients in our study received > 50 Gy. First, toxicity risk is quantified by the dose and volume delivered to organs at risk. We observed a positive correlation between radiation dose and the incidence of TRAEs, as escalating the prescription dose tends to increase the actual dose delivered to surrounding organs at risk. Furthermore, a higher T-stage correlated with both elevated dosimetric parameters (e.g., lung V20/V30) and increased AE incidence. This is likely because a larger initial tumor volume implies greater irradiation of normal tissues like the lung, heightening toxicity risk even after treatment response. Second, the anatomical proximity of tumors to critical organs is an inherent constraint, difficult to fully circumvent through planning optimization. Consequently, centrally located lesions or those near the esophagus or heart carry an intrinsically higher toxicity risk. Such spatial relationships directly shape distinct normal tissue irradiation patterns and risk profiles across patients. Additionally, a patient's general condition significantly influences treatment tolerance. KPS and the presence of brain metastases were independently associated with adverse events, suggesting that patients in poorer general condition at baseline are more susceptible to treatment-related toxicities at comparable therapeutic intensity. Therefore, safety management should shift from reliance on population-level data toward individualized, precision assessment. In practice, radiotherapy intensity must be carefully weighed based on a comprehensive patient evaluation to identify the most suitable personalized regimen, thereby optimizing the efficacy-safety balance in the immunotherapy era. Our study has several limitations. First, as a retrospective study, patient treatment regimens were heterogeneous. Although we attempted to control for confounders, residual confounding from unmeasured factors may still influence the results. Second, the limited sample size compromised the complete balance of baseline characteristics among treatment subgroups and reduced the power of the statistical analyses. Additionally, the retrospective nature of this study limited our ability to verify the consistent application of certain precise radiotherapy techniques, such as respiratory motion management, which may have affected the accuracy of treatment delivery evaluation.

Thoracic radiotherapy studies in ES-SCLC have evolved from solely focusing on local control to integration with systemic therapy. Advances in radiotherapy technology enable clinicians to tailor regimens based on tumor size, metastatic status, and baseline status, contributing to the gradual optimization of thoracic radiotherapy strategies in ES-SCLC. Future directions should expand precision radiotherapy techniques, develop novel immunotherapy combinations, and personalize treatments based on molecular profiles. Further exploration is required to investigate the dynamic balance between radiotherapy strategies and toxicity management, aiming to maximize survival benefits in the context of immunotherapy.

## Conclusion

An integrative evaluation of efficacy and safety demonstrates that thoracic radiotherapy delivered after CIT is superior to concurrent radiotherapy with CIT. For patients receiving concurrent thoracic radiotherapy, a low-tBED regimen may yield superior prognostic outcomes compared to a high-tBED regimen. Furthermore, the analysis revealed that factors such as sex, KPS, tBED, and brain metastasis status were significantly associated with TRAEs. Meanwhile, age, KPS score, tBED, and baseline distant metastasis status were significantly correlated with patient prognosis.

## Compliance with ethical standards

### Guarantor

The scientific guarantor of this publication is Shuanghu Yuan.

### **Ethical** approval

Institutional Review Board approval was obtained.

## CRediT authorship contribution statement

**Hao Zhou:** Data curation, Formal analysis, Investigation, Methodology, Validation, Writing – original draft. **Huan Zhao:** Data curation, Methodology. **Shuming Shi:** Data curation, Writing – review & editing. **Tao Hu:** Data curation, Writing – review & editing. **Li Li:** Data curation, Writing – review & editing. **Shuai Wang:** Investigation, Writing – review & editing. **Zhe Zhang:** Investigation, Writing – review & editing. **Shuanghu Yuan:** Conceptualization, Funding acquisition, Resources, Supervision, Validation, Writing – review & editing.

## Informed **consent**

This study was approved by the Ethics Committee of the Shandong Cancer Hospital and Institute (ethics approval number: SDTHEC2020004042). This study received a waiver of informed consent from the institutional ethics committee due to its retrospective design and the use of fully anonymized data. All procedures performed in studies involving human participants were in accordance with the ethical standards of the institutional and/or national research committee and with the 1964 Helsinki declaration and its later amendments or comparable ethical standards.

## Funding

This work was supported by the National Natural Science Foundation of China (NSFC82073345), the National Natural Science Foundation of China (NSFC82473235), Natural Science Innovation and Development Joint Foundation of Shandong Province (ZR202209010002), Jinan Clinical Medicine Science and Technology Innovation Plan (202019060), and Taishan Scholars Program to Shuanghu Yuan.

## Declaration of competing interest

The authors declare the following financial interests/personal relationships which may be considered as potential competing interests: [The authors declare that the research was conducted in the absence of any commercial or financial relationships that could be construed as a potential conflict of interest.].
